# Esophageal actinobacillosis mimicking a diverticulum in a cow: a case report

**DOI:** 10.1099/acmi.0.000819.v4

**Published:** 2024-09-06

**Authors:** Rahul Kumar, Gulshan Kumar, R. P. Pandey

**Affiliations:** 1 Department of Veterinary Pathology, College of Veterinary Science & Animal Husbandry, DUVASU: Uttar Pradesh Pandit Deen Dayal Upadhyaya Pashu Chikitsa Vigyan Vishwavidyalaya Evam Go Anusandhan Sansthan, Mathura-281001, Uttar Pradesh, India; 2 Department of Veterinary Surgery and Radiology, College of Veterinary Science & Animal Husbandry, DUVASU: Uttar Pradesh Pandit Deen Dayal Upadhyaya Pashu Chikitsa Vigyan Vishwavidyalaya Evam Go Anusandhan Sansthan, Mathura-281001, Uttar Pradesh, India

**Keywords:** actinobacillosis, cattle, esophagus, histopathology, skiagram, surgery

## Abstract

Present case report describes a case of an atypical oesophageal actinobacillosis in an adult cow presented to the university hospital with a history of inability to drink and swallow. Clinical examination revealed a five-inch swelling in the jugular groove. Skiagram revealed the presence of a small and slightly radio opaque round growth. Exploratory surgical excision of the growth was adapted as palliative treatment and the extirpated tissue was fixed in 10% buffered formalin. Histopathological examination revealed pyogranulomatous inflammation with radiating eosinophilic club shaped bodies surrounding small colonies of coccobacilli. Gram and Ziehl-Neelsen stains confirmed the presence of Gram-negative and non-acid-fast coccobacilli. Histopathology confirmed the pathognomonic lesion and proved to be a modality of choice for pathologists to reach at a diagnosis of atypical oesophageal actinobacillosis in a cow. After the exhaustive search of relevant literature on atypical actinobacillosis, the authors claim this to be the second report of oesophageal actinobacillosis worldwide.

## Introduction

*Actinobacillus ligneresii* is a Gram-negative coccobacillus often found as a commensal in the oral cavity [1,2] and anterior part of the digestive tract of domestic animals [3, 4]. Trauma, erosion, ulcer and penetrating lesions induced by hard fibrous feed causes a breach in the integrity of mucosal epithelium establishing infection [5, 6]. The causes are sometimes secondary and also iatrogenic [7, 8]. Sporadically, it generally affects the soft tissue of animals and the typical pyo-granulomatous lesions are found in tongue (wooden tongue), rumen and reticulum. However, atypical lesions are reported as subcutaneous granulomas [9,10], non-woody tongue [11], in lymph nodes [2,12], generalized actinobacillosis involving heart, kidneys, cervical lymph nodes [13] and other tissues of the head, pharynx, chest, flank, stomach, omentum and limbs [4, 14]. To the best of author’s information, only one case of this disease affecting oesophagus is reported in the veterinary literature [12]. An unusual clinical presentation, radiological assessment, surgical treatment and pathological findings of actinobacillosis are reported here, where a large nodule involving the oesophagus, resulting into apprehensive and regurgitative gastric disorder, was observed in a cow.

## Case presentation

**Fig. 1.**
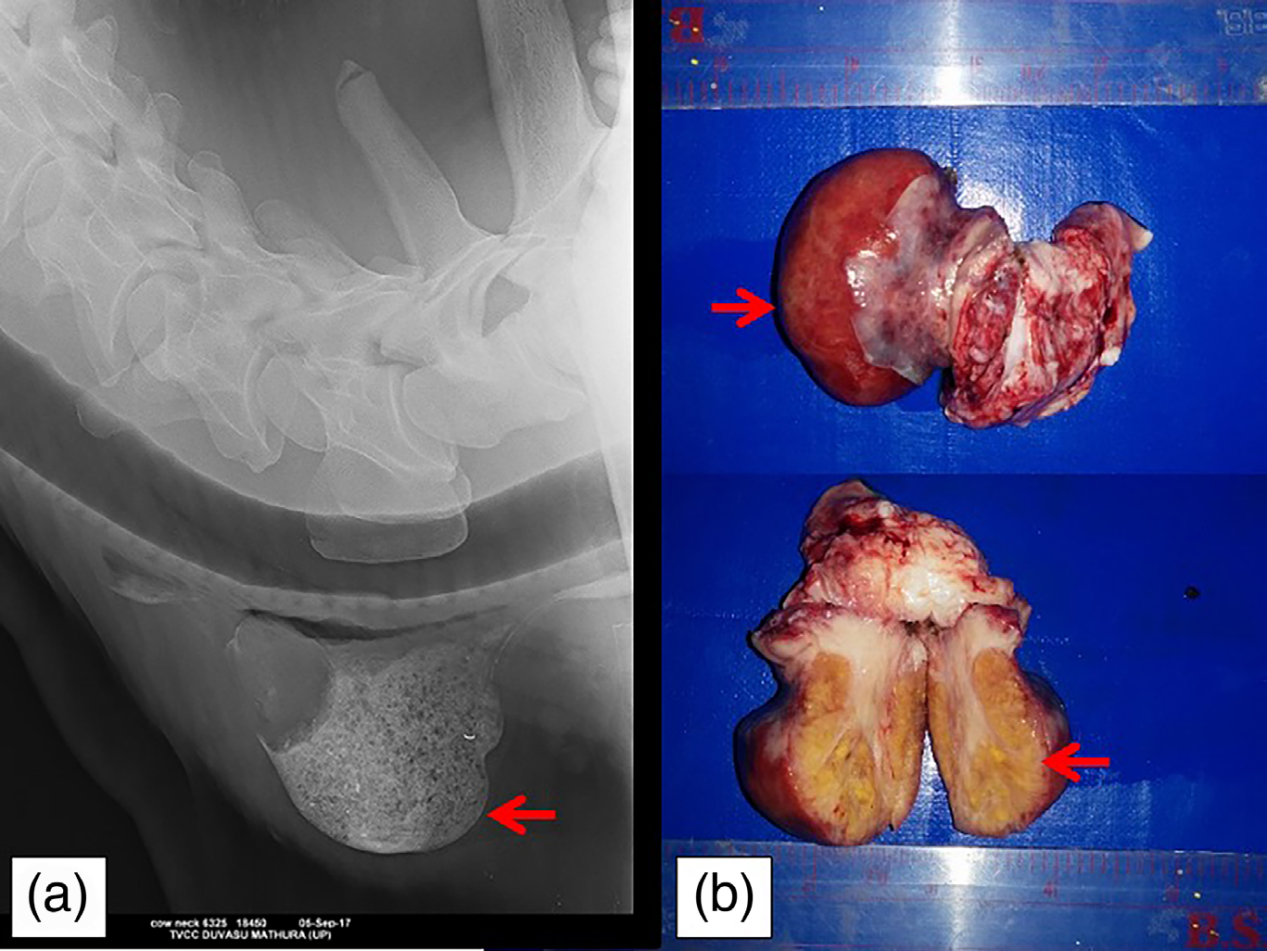
Radiographic appearance of the jugular groove, latero-lateral projection. (a) Mass is evident in the esophagus (arrows). (b) The extirpated mass having yellow coloured parenchyma (sulphur granules).

An adult cow with a history of difficult breathing, inability to drink water or swallow feed was brought to the Teaching Veterinary Clinical Complex (TVCC), DUVASU, Mathura. These signs increased in severity over a period of 3 months. During clinical examination, the attending clinicians observed that drinking led to regurgitation. There was a swelling in about 5-inch area in the mid cervical jugular groove. Probang could not be passed beyond this part. Lateral skiagram showed dilatation and presence of a small slightly radio opaque round lesion. Oesophageal lavage helped to clear the accumulated ingesta and subsequent radiography revealed a diverticulum and a tumor on the esophageal wall (Fig. 1a). The clinical suspicion and the anatomic location of the growth was confirmed by radiography.

An exploratory surgical excision of the growth was decided as palliative therapy. After intravenous rehydration with isotonic saline solution (0.9% NaCl) administered through a jugular vein catheter (10 ml kg^−1^ hr^−1^), esophagotomy was done in left lateral recumbency under xylazine sedation (0.05 mg kg^−1^ IV) and local anaesthesia (2% lidocaine hydrochloride). The affected part of oesophagus was exposed through a 7-inch-long incision along the mid cervical jugular groove over the swelling, oesophagus was entered, its lumen exposed, and the obstructive mass, which was pedunculated and attached to the oesophageal wall, was located. The mass including the part of oesophagus where it had its base was reached, and after excising the diverticulum part as far as possible maintaining the lumen patency, esophagotomy incision was closed by apposing the mucosa using a simple continuous pattern with absorbable sutures (3–0 Vicryl) and the muscular layer was sutured using a simple interrupted pattern with absorbable sutures (2–0 Vicryl). Skin incision was partially left open to allow post-operative drainage and was loosely approximated using non-absorbable sutures (Nylon 2–0). Post operatively, 48 h fluid therapy (isotonic saline at a rate of 500 ml hour^−1^) followed by allowing oral intake of water and soft green fodder was recommended. Strepto-Penicillin (2.5 gm IM twice a day), multivitamin injections (B-complex, 1 ml IM) and ketoprofen injection (3 mg kg^−1^ IM) all once daily for 7 days were administered. The multivitamin included B1, B6, and B12, essential for supporting metabolic function and healing. Surgical wound care consisted of cleaning and applying povidone iodine ointment and using permethrin-based fly repellent spray till complete healing.

## Results and discussion

Exploratory surgical excision of the mass and no reoccurrence suggested a valid option for the treatment of *Actinobacillus* pyogranulomas as adopted by [15]. The surgical excision followed by no remission after 1 year of surgery is significant, because the resolution was achieved without the use of iodine, suggested to be the treatment of choice for *Actinobacillosis* [16].

The respiratory and digestive symptoms shown by the present case are due to the pressure on the trachea and oesophagus causing obliteration of their lumen, respectively. The radiographic picture helped in assessing the anatomic location of the mass and thereby adapting an appropriate surgical procedure. The mass was differentiated from other obstructive causes viz. tumour, abscess, cyst, hematoma, allergic, mycotic, tubercular, actinobacillar or parasitic granulomas.

Grossly, the mass was pedunculated, weighing 37 g, measured 4.5 cm × 5.5 cm having an intensely red surface. The stalk of the mass was soft whereas the mass was hard to cut. The cut surface revealed yellow foci (sulphur granules) of 1–2 cm in a densely packed fibrous stroma (Fig. 1b). The surgically removed mass was fixed in 10% NBF and sent to the Department of Veterinary Pathology for histopathological examination.

**Fig. 2.**
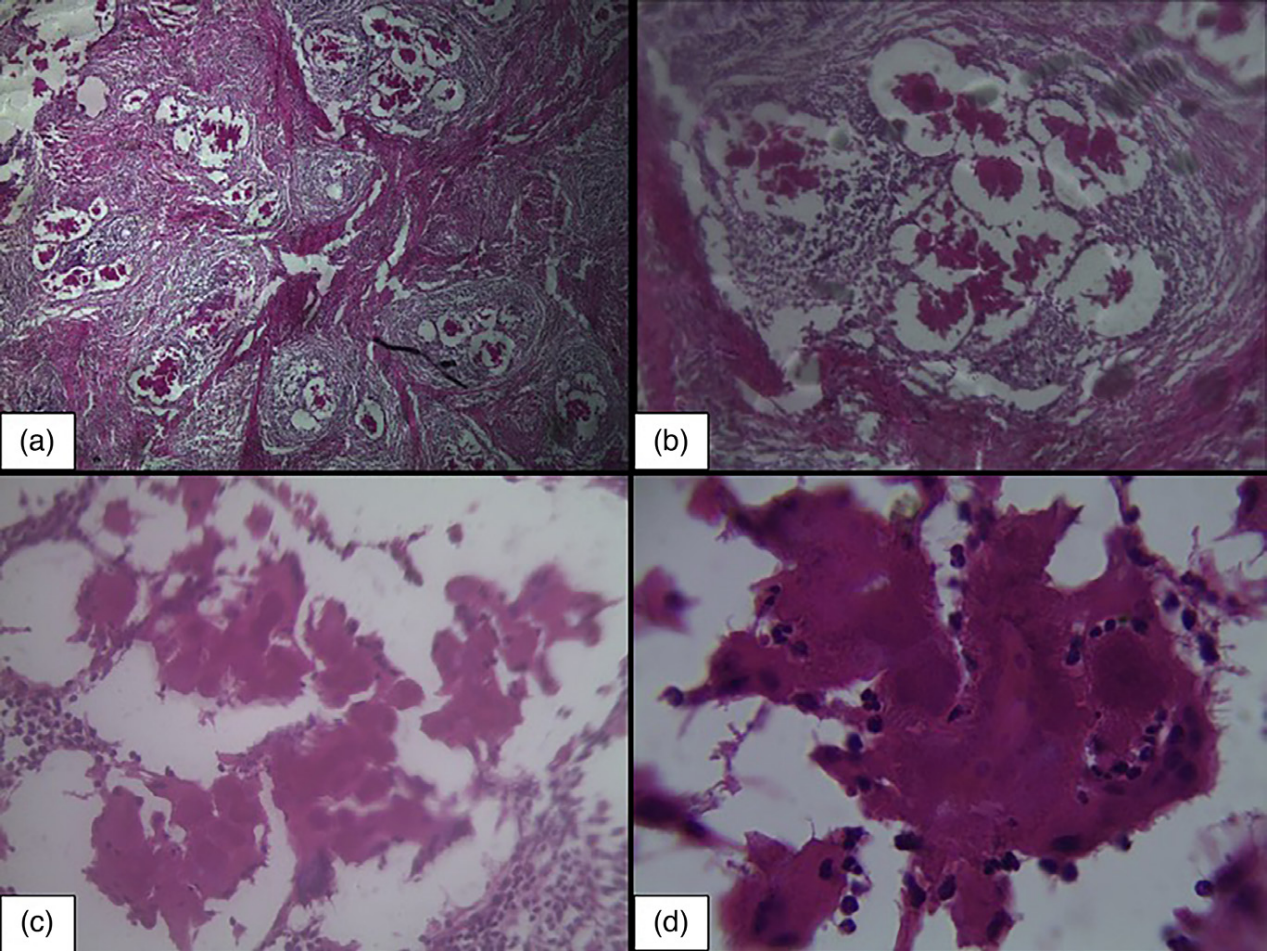
Oesophageal actinogranuloma. (a) The mass is composed of fibrous tissue and multiple confluent pyogranulomas. H and E stain, ×40. (b) Actinobacillar pyogranuloma. Bacterial colonies are surrounded by eosinophilic club-like bodies, neutrophils, and large macrophages. H and E stain, ×100. (c) and (d) showing the details of actinogranuloma at higher magnifications of H and E stain, 400× and 1000× respectively.

Microscopic examination of formalin fixed, paraffin embedded and H&E stained sections from the extirpated mass revealed that it was composed of fibrous tissue and confluent pyogranulomatous inflammatory abscesses (Fig. 2a, b). There were multiple tiny microgranulomas (Fig. 2a, b) containing mixed population of inflammatory cells (Fig. 2c, d) viz. lymphocytes, few neutrophils, plasma cells and macrophages encased in a dense fibrous connective tissue layer (Fig. 2b). The microgranulomas contained actinobacilli located at the centre with radiating eosinophillic clubs popularly known as Splendore-Hoepelli material (Fig. 2d).

Affection of exclusively soft tissues like tongue and regional lymph nodes in actinobacillosis has also been reported previously [4,6, 11, 17, 18]. Gram and Ziehl-Neelsen stains for acid-fast bacteria were then carried out which showed Gram-negative and non-acid-fast coccobacilli. Coccoid organisms at the centre of pyogranulomas were also reported by [19]. Based on the above observations, a histopathological diagnosis of an atypical case of oesophageal actinobacillosis probably caused by *Actinobcillus ligneresii* was made.

Histopathology has been proved to be the best modality for differentiation among tumour, abscess, cyst, hematoma, allergic, mycotic, tubercular, actinobacillar or parasitic granulomatous lesions. This case was diagnosed as actinobaccilosis primarily based on pathognomonic histopathological lesions, i.e. Splendore-Hoepelli material within pyogranulomas, strongly suggesting *Actinobacillus lignieresii* infection. While histopathological evidences are substantial for diagnosing actinobaccillosis, it must be supported with isolation and identification of the bacteria, its cultural, morphological, serological and molecular characteristics. The Gram-negative and non-acid-fast staining properties, size and shape of the bacteria ruled out the possibility of tubercular (Mycobacteria and fungi), pyogranulomatous (*Actinomyces* and *Staph. aureus*) or other microorganisms. Absence of bacterial culture and subsequent identification represents a limitation in our study. However, in this case, obtaining a culture was not feasible due to the presentation and handling of the sample.

The potential route of infection leading to oesophageal actinobacillosis in this case could be attributed to: 1) direct inoculation through trauma or injury to the oral cavity or oesophageal mucosa caused by rough or fibrous feed creating an entry point for *Actinobacillus lignieresii*, allowing the bacteria to penetrate and establish an infection in the oesophageal tissue. This is the common mode of entry for other forms of actinobacillosis, such as wooden tongue. 2) Secondary spread from the infection originating in the oral cavity or another primary site and subsequently spread to the oesophagus through lymphatic or hematogenous routes. Given the organism’s ability to cause localized and disseminated pyogranulomatous lesions, secondary spread to the oesophagus is a plausible route of infection. 3) Another potential route of infection could be iatrogenic, where procedures involving the oral cavity or oesophagus might inadvertently introduce the bacteria. However, there is no specific history of such procedures in the current case. Given the cow’s clinical presentation, the most likely route of infection appears to be direct inoculation through mucosal breaches caused by rough feed, leading to localized oesophageal actinobacillosis.

To the best of the author’s knowledge and based on an extensive literature review, oesophageal actinobacillosis remains an exceedingly rare condition with very few reported cases in veterinary literature. The case presented by Mortimer in 1962 [5] appears to be the earliest and only documented instance. This current report aims to contribute additional insights into this rare manifestation of actinobacillosis.

## Conclusion

In conclusion, this case highlights an atypical presentation of oesophageal actinobacillosis. The combination of clinical, radiographic, and histopathological findings supports the diagnosis and effective management of this rare condition. While histopathological findings strongly support the diagnosis of actinobacillosis, the absence of bacterial culture remains a limitation. This highlights the importance of combining histopathological examination with culture and molecular methods to achieve a more definitive diagnosis. Despite this limitation, the successful surgical intervention and lack of recurrence in this case underscore the importance of considering actinobacillosis in differential diagnoses for oesophageal masses in cattle. Based on the available follow-up data, the absence of recurrence after 1 year post-surgery is encouraging and suggests a positive outcome. However, it is important to interpret these findings with caution due to the limited duration of follow-up. Long-term monitoring is essential to confirm the efficacy and durability of the surgical intervention for oesophageal actinobacillosis.

## Abbreviations

DUVASU: DeenDayal Upadhyay Veterinary and Animal Science University
H&E stain: hematoxylin and eosin stain
IM: intramuscular
IV: intravenous
NBF: neutral buffered formalin
TVCC: Teaching Veterinary Clinical Complex
